# Deformation Induced Structure and Property Changes in a Nanostructured Multiphase CrMnFeCoNi High-Entropy Alloy

**DOI:** 10.3390/nano13050924

**Published:** 2023-03-02

**Authors:** Benjamin Schuh, Inas Issa, Timo Müller, Thomas Kremmer, Christoph Gammer, Reinhard Pippan, Anton Hohenwarter

**Affiliations:** 1 Erich-Schmid-Institute of Materials Science, Austrian Academy of Sciences, Jahnstraße 12, 8700 Leoben, Austria; 2 Department of Materials Science, Montanuniversität Leoben, Jahnstraße 12, 8700 Leoben, Austria; 3 Chair of Nonferrous Metallurgy, Montanuniversität Leoben, Franz-Josef-Straße 18, 8700 Leoben, Austria

**Keywords:** severe plastic deformation, high-pressure torsion, high entropy alloys, cantor-alloy, nanocrystalline

## Abstract

A nanocrystalline CrMnFeCoNi high-entropy alloy produced using severe plastic deformation using high-pressure torsion was annealed at selected temperatures and times (450 °C for 1 h and 15 h and at 600 °C for 1 h), causing a phase decomposition into a multi-phase structure. The samples were subsequently deformed again by high-pressure torsion to investigate the possibility of tailoring a favorable composite architecture by re-distributing, fragmenting, or partially dissolving the additional intermetallic phases. While the second phase in the 450 °C annealing states had high stability against mechanical mixing, a partial dissolution could be achieved in the samples subjected to 600 °C for 1 h.

## 1. Introduction

The most prominent high-entropy alloy (HEA) investigated over the last 20 years is the equiatomic CrMnFeCoNi alloy, also known as Cantor-alloy, which exhibits excellent ductility, a high work-hardening rate, and good fracture toughness down to cryogenic testing conditions [1,2]. In the first studies, it was assumed to be thermodynamically stable [3,4], but in recent years it has been proven that heat treatments below temperatures of 800 °C result in phase decomposition [5,6,7,8]. Depending on the grain size, the annealing results in increased hardness but also in a deterioration of the ductility [7]. However, the presence of second phases could also be advantageous to the ductility of nanocrystalline (NC) metals, following the premise that they can restore the usually low work-hardening rate [9].

In the recent past, a nanocrystalline (NC) CrMnFeCoNi alloy was annealed to achieve a multi-phase state, and it was demonstrated via nanoindentation that the second phases are prone to deformation-induced dissolution [10]. This result sparked the idea to tailor the microstructure of a multi-phase NC CrMnFeCoNi alloy via severe plastic deformation (SPD) by applying higher deformation degrees to considerably larger material volumes compared to nanoindentation. Tuning the size of the second phase particles by partial dissolution and their SPD-induced re-distribution could be a pathway to overcome the ductility issue in NC metals. Additionally, mechanically induced instability by the dissolution of phases could also have implications for the material behavior during wear, contact fatigue, or low–cycle fatigue conditions where often deformation degrees are large and can, for example, trigger local softening and crack initiation [11]. Such trends regarding local softening or hardening can also be captured by the following study.

## 2. Materials and Methods

For the synthesis of the CrMnFeCoNi HEA, high-purity elements (≥99.9 wt%) were arc melted and drop cast under an Ar atmosphere, and the ingots were homogenized at 1200 °C for 48 h. Afterward, disks (8 mm in diameter and 0.8 mm in thickness) were manufactured by electric discharge machining and deformed via high-pressure torsion (HPT). By applying this technique, the disk-shaped specimens are deformed by shear, and the applied shear strain, γ, can be calculated as follows:

$$\gamma =\frac{2\pi r}{t}\cdot n$$

where *r* is the radius of the disk measured from the disk center; *t* is the thickness of the sample; and *n* is the number of rotations. More details on the principles of the applied SPD technique and the experimental setup can be found elsewhere [12,13].

The samples were deformed up to 5 rotations at a nominal pressure of 7.8 GPa with a rotational speed of 0.2 rotations per minute. This high amount of applied strain led to a fairly pronounced homogenization of the hardness in large parts of the sample. The homogenization can be interpreted as the occurrence of an equilibrium NC microstructure [14]. The microstructure after HPT deformation at room temperature features an equilibrium grain size of 50 nm, as was already established in a previous study [7]. Then the specimens were annealed at 450 °C for 1 h to 15 h or at 600 °C for 1 h, which led to the formation of various second phases. The annealed samples were then again subjected to HPT processing to see the influence of deformation on the second phase. They were deformed up to 100 rotations with the same nominal pressure and rotational speed as before. 

Microstructure investigations were performed utilizing a scanning electron microscope (SEM, Zeiss LEO-1525 Carl Zeiss GmbH, Oberkochen, Germany) as well as transmission electron microscopes (TEM, JEOL 2100F, JEOL, Akishima, Japan) and Thermo ScientificTM Talos F200X G2 (ThermoFisher Scientific, Hillsboro, OR, USA). Microstructural images of the 450 °C annealing states were taken in scanning TEM (STEM) mode and brightfield-mode. Phase analysis was performed by energy dispersive X-ray (EDX) mapping. For the coarser-grained 600 °C annealing state, images were taken by back-scattered electron (BSE) imaging, and phase analysis was performed both with the help of an electron back-scatter detector (EBSD) as well as point analysis of BSE images. Complementary X-ray diffraction experiments (XRD) were performed with a D2 phaser using Co-K_α_ radiation (Bruker Corporation, Billerica, MA, USA). For mechanical tests, Vickers microhardness measurements were conducted with a microhardness tester, Micromet 5104 from Buehler (Düsseldorf, Germany), at a load of 1000 gf and dwell times of 15 s. For tensile tests, two dogbone specimens per HPT disk were prepared via electrical discharge machining. The gauge length of the tensile sample is located at a radius of 2 mm and therefore well within the microstructural homogeneous saturation area of the HPT disk. Tensile samples had a nominal cross-sectional area of the diameter of 500 × 500 μm² and a gauge length of 2.5 mm and. Tests were then performed utilizing a tensile testing machine from Kammrath and Weiss (Schwerte, Germany) at room temperature with a 2 kN load cell and a crosshead speed of 2.5 µm/s. At least 2 samples were tested per condition.

## 3. Results 

### 3.1. Initial Microstructure

**Figure 1:**
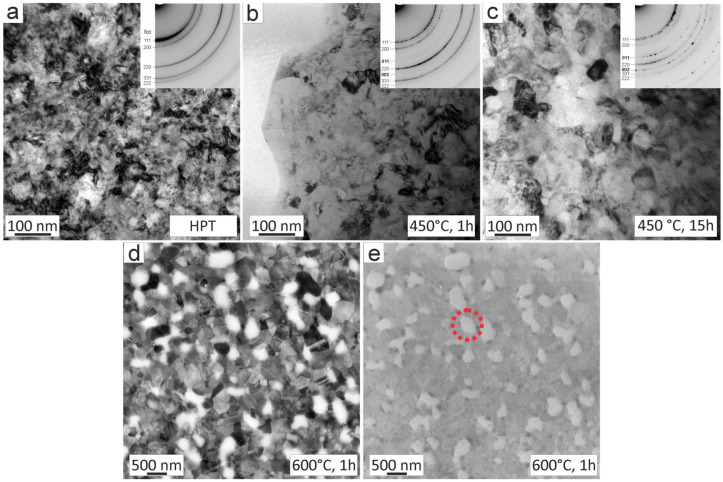
Overview of the investigated microstructures. (**a**) SPD-processed state by HPT. (**b**) Annealed specimen at 450 °C for 1 h. (**c**) Annealed specimen at 450 °C for 15 h. (**d**) Annealed state at 600 °C for 1 h investigated with SEM using a back-scatter electron detector. (**e**) Annealed state at 600 °C. Same position as in (**d**) using a secondary electron detector. One example of the additional phase is marked with a circle. Please note that (**a**–**c**) are TEM brightfield images whereas (**d**,**e**) is made with SEM.

Figure 1a depicts the NC microstructure of the HPT-deformed sample before annealing, where an average equilibrium grain size of approximately 50 nm was measured. The corresponding diffraction pattern, in the inset of Figure 1a, reveals a single-phase disordered face-centered cubic (fcc) crystal structure. In Figure 1b,c, the microstructure after annealing for 1 h and 15 h at 450 °C is presented.

At this stage, grain growth is still very limited; however, the corresponding diffraction patterns in the inset clearly show the occurring phase decomposition through the presence of additional spotty diffraction rings. The additional rings, however, are very weak. The ones indexed in Figure 1b,c can be ascribed to a bcc-phase (bold indices), most probably to a Cr-rich phase according to a former publication [7]. Heat treatments at 600 °C for 1 h lead to a distinctive grain coarsening. This is shown in an SEM image in Figure 1d using a back-scatter electron detector, which is capable of revealing the matrix. Using the secondary electron detector and inspecting the same position, the presence of an additional phase can be confirmed (Figure 1e).

**Figure 2:**
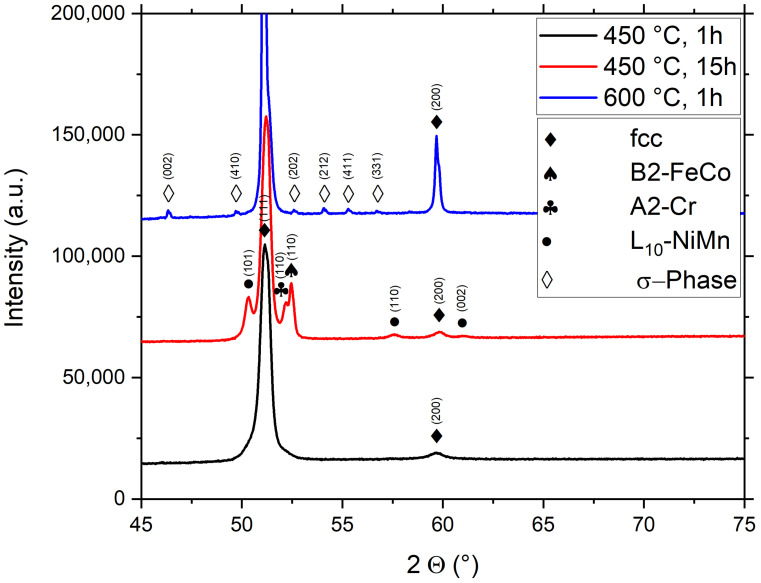
XRD patterns of the HPT-deformed Cantor-alloy after HPT-processing and subsequent annealing at 450 °C for 1 h and 15 h as well as at 600 °C for 1 h. The theoretical reference peaks in this figure and the following ones are found in the Crystallography Open Database (B2-FeCo: COD 9004229, A2-Cr: COD 5000220, L10-NiMn: COD 1523522, σ-phase: COD 2106166). For the fcc-phase, a lattice constant of 3.6 A was taken according to reference [15].

The crystallographic phases are further investigated with the XRD data shown in Figure 2. While it has been established via atom probe tomography measurements that in NC samples, 1 h of annealing at 450 °C leads to the formation of second phases [7], their volume fraction is not large enough to be visible via XRD measurements; see Figure 2. However, prolonged annealing at 450 °C for 15 h leads to a significant formation of second phases. The phase decomposition results in the formation of a microstructure with four different phases, which can be inferred from the corresponding XRD pattern. Due to the limited number of peaks, some of these phases cannot be unambiguously identified from the diffractograms; however, their presence is indicated in the diffractograms in combination with knowledge documented in other experimental studies [5,6,8]. These four phases are the original high-entropy phase with an fcc crystal structure, an A2-phase (Cr-rich), a B2-phase (FeCo-rich), as well as an L_10_-phase (NiMn-rich). After annealing at 600 °C a multi-phase microstructure has formed, consisting of the high-entropy fcc phase and a σ-phase.

### 3.2. Post-Deformation of Multiphase Microstructures

**Figure 3:**
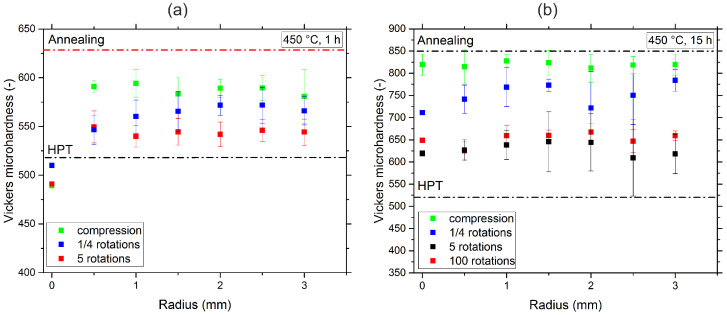
Hardness evolution of the CrMnFeCoNi alloy during HPT deformation of the samples previously annealed at 450 °C for 1 h (**a**) and 15 h (**b**).

The samples were annealed at 450 °C for 1 and 15 h and subsequently deformed via HPT. The hardness measurements after this treatment are shown in Figure 3a,b. All samples underwent compression and then subsequently up to five rotations in the HPT, resulting in an applied shear strain, γ, of approximately 170 for a sample radius of 3.5 mm. In the case of the 450 °C, 15 h sample (Figure 3b), up to 100 rotations were applied to see if extremely high strains led to a further change in the second phase behavior. In Figure 3a the hardness of the as-annealed 450 °C, 1 h sample is 630 ± 2 HV1. After compression, the hardness scatters between 551 HV1 and 600 HV1, which is because HPT samples are not undergoing a homogenous compressive strain [13]. At the edges of the samples, higher compressive strains are usually realized. As shear strain is applied, the hardness decreases to about 540 ± 12 HV1 and then levels off into a plateau at a shear strain γ of more than 30. For comparison, the hardness of the HPT-deformed single-phase sample before annealing is about 519 ± 3 HV1.

The sample annealed for 15 h at 450 °C shows a similar behavior; see Figure 3b. The initial hardness is significantly higher, 852 ± 8 HV1. As a result, the sample is fairly brittle, which leads to crack formation during HPT deformation. Therefore, large deviations in hardness across the radius are present. Nonetheless, the same trend as in the 1 h sample can be observed: Upon deformation, the hardness of the sample decreases and then plateaus even after 100 rotations. 

**Figure 4:**
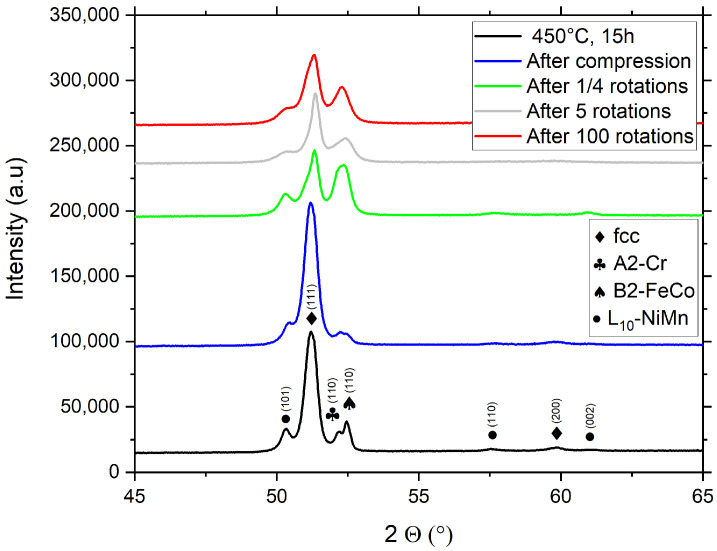
XRD-patterns of the annealed HPT-deformed Cantor alloy (450 °C for 15 h) subjected to different degrees of deformation.

Figure 4 depicts XRD measurements of the deformed materials after 450 °C, 15 h of annealing, and subsequent deformation. The 450 °C, 1 h annealing state is not shown, since even in the annealed state, no XRD signal from the second phases could be found (Figure 2). During HPT deformation, peak broadening occurs due to the abundant formation of lattice defects, which cause an overlap of the B2-FeCo and the A2-Cr phases. From the peak intensities, it is clear that even under the most severe deformation condition of 100 rotations in the HPT, full re-dissolution of the second phases has not been achieved.

**Figure 5:**
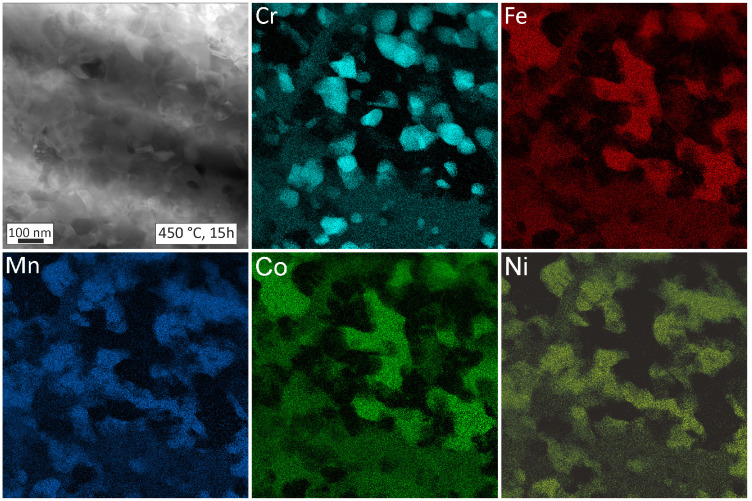
STEM image of the 450 °C, 15 h as-annealed state and corresponding elemental mapping of Cr, Fe, Mn, Co and Ni.

**Figure 6:**
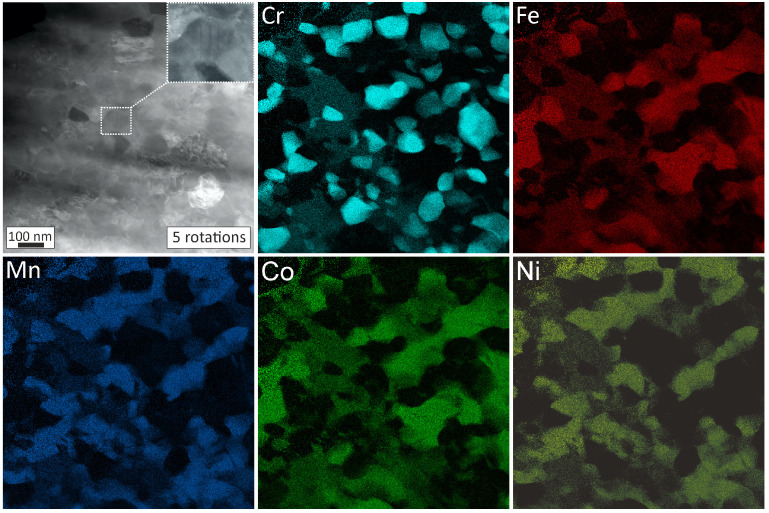
STEM image of the 450 °C, 15 h annealed state after deformation for 5 rotations by HPT and corresponding elemental mapping of Cr, Fe, Mn, Co and Ni.

The slight change in peak position of the (111) peak of the high-entropy fcc phase to higher 2Θ angles after deformation indicates that re-dissolution to some degree occurs, leading to a change in the lattice constant of the phase caused by a change in the phase chemistry. However, quantitative phase analysis of the XRD-data is difficult due to overlapping peaks as well as peak intensity changes based on the formation of a shear texture during HPT-deformation [16]. Thus, in order to study the amount of fragmentation and re-dissolution in detail, an additional microstructural study via TEM was (performed see Figure 5 and Figure 6).

In Figure 5, STEM images of the 450 °C, 15 h state after annealing can be seen combined with elemental maps indicating the elemental distribution of Cr, Mn, Fe, Co, and Ni, respectively. Especially, Cr, Mn, and Fe are of interest as they represent the main constituents of the second phase. In Figure 6, STEM images of the same annealed state (450 °C, 15 h) but additionally deformed for 5 rotations are presented. The elemental mappings allowed for a quantitative analysis of the phase distribution; these results are summarized in Table 1. For that, a Ni-Mn phase was assigned if Mn was larger than 35% (the same result is obtained if the condition is Ni > 35%). A Fe-Co phase was assigned if Fe > 35% (almost the same result is obtained if the query is Co > 35%). The Cr-rich phase (A2-Cr) was assigned if Cr > 40%. Surprisingly, unlike what was reported in reference [10], the re-dissolution after HPT deformation of the annealed samples is minimal. However, some microstructural changes are nonetheless visible; the microstructure after deformation is more blurry, which is due to the reintroduction of non-equilibrium grain boundaries [17,18]. Furthermore, in the STEM image (Figure 6), deformation twins can be seen induced by plastic deformation. 

**Figure 7:**
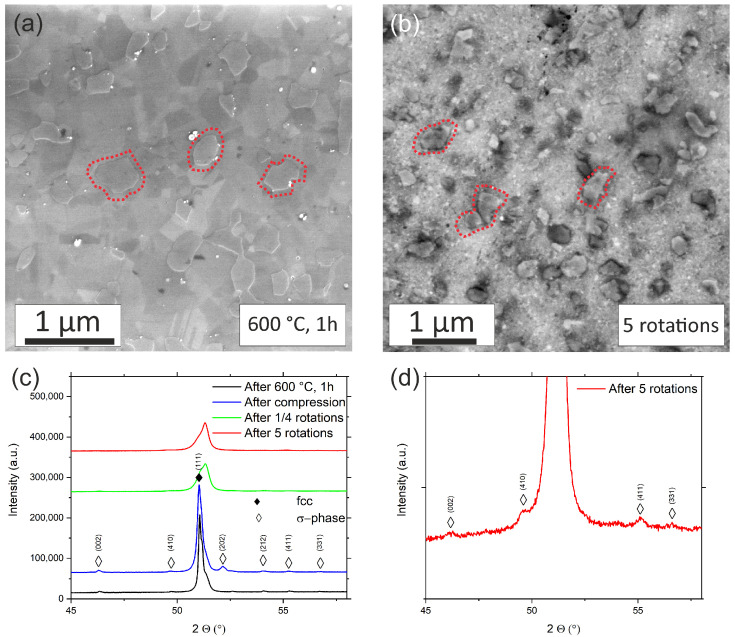
(**a**) BSE micrograph of the annealed 600 °C, 1 h specimen. (**b**) Microstructure after deformation for 5 rotations. For clarity some examples of the σ-phase are marked in both images in red (**c**) XRD data of the as-annealed state and after deformation. (**d**) detail of the XRD-data of (**c**) after 5 rotations. It can be seen that the σ-phase is still present after deformation.

Annealing at 600 °C, 1 h causes structural coarsening and the formation of the σ-phase (Figure 7a). In this case here the volume fraction of the σ-phase was characterized by point-analyses of multiple BSE-images. The average volume fraction of the σ-phase is 19.5 ± 1%. Following deformation for 5 rotations, the high-entropy matrix features NC grains due to the severe grain refinement imposed by HPT-deformation. The refinement or dissolution of the σ-phase particles is less pronounced (Figure 7b).

More importantly, due to the high amount of applied shear strain, a significant fraction of the σ-phase has been fragmented and possibly dissolved into the matrix. After 5 rotations the average volume fraction of the σ-phase is 13.6 ± 2 vol%. XRD confirms the presence of the σ-phase and the decrease of its volume fraction during HPT deformation, indicated by the pronounced change in the peak-height (Figure 7c,d). A closer look at the pattern of the specimen subjected to five rotations clearly proves that the σ-phase has not been fully resolved. In addition, it must also be granted that a strong fragmentation could have the same effect on the XRD-pattern.

**Figure 8:**
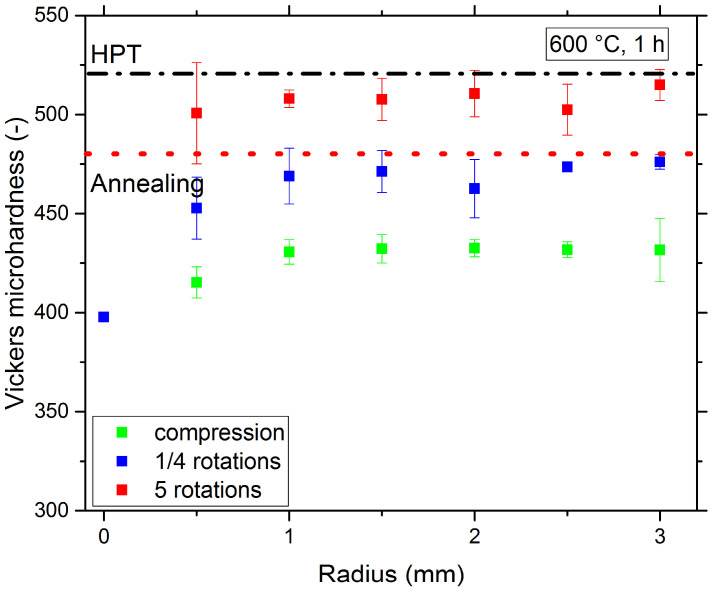
Hardness evolution during deformation of the 600 °C, 1 h annealed sample as a function of sample position and number of rotations.

Regarding the mechanical response, see Figure 8, the initial hardness of 480 ± 3 HV1 after annealing at 600 °C, 1 h, is lower than in the HPT-deformed material, which is due to the onset of grain growth for this temperature. When subsequently compressive strain is applied, the hardness of the samples decreases further to 425 ± 8 HV1. This phenomenon can be explained by the reversal of the so-called “hardening by annealing” effect [19]. 

When the NC samples are initially annealed, their hardness increases not only due to the formation of second phases but also since the grain interior is starved of dislocation sources due to defect annihilation. Once plastic deformation needs to be realized (e.g., for performing a hardness test), again, higher stress levels are needed. This effect is profoundly discussed in the literature and rationalized in terms of a change in the dislocation absorption or annihilation processes at grain boundaries [19].

In the case of the annealed and subsequently deformed samples, mobile defects are reintroduced into the structure. Due to their mobility, these defects reduce the overall hardness of the alloy. If more deformation is applied, the hardness increases again and then saturates at the same level as the initial HPT-deformed sample, 507 ± 12 HV1 (annealed and deformed) vs. 519 ± 34 HV1 (HPT only). This is due to the fact that with higher deformation, grain refinement down to the initial NC grain size is again achieved, which results in a similar hardness. This behavior clearly indicates that the large σ-phase particles only have a low contribution to the hardness, since they are: (I) mostly located at grain boundaries and triple junction and hence they do not represent obstacles to intragranular dislocation motion and (II) their volume fraction is too low to effectively constrain the surrounding softer matrix, which would lead to a composite-type hardening. In contrast to this restoration of the original hardness level, the 450 °C anneals subjected to further deformation lead to a hardness level between the annealed and the pure HPT-state, because the hardness is not only determined by the grain size but also by the presence of additional intermetallic phases. 

**Figure 9:**
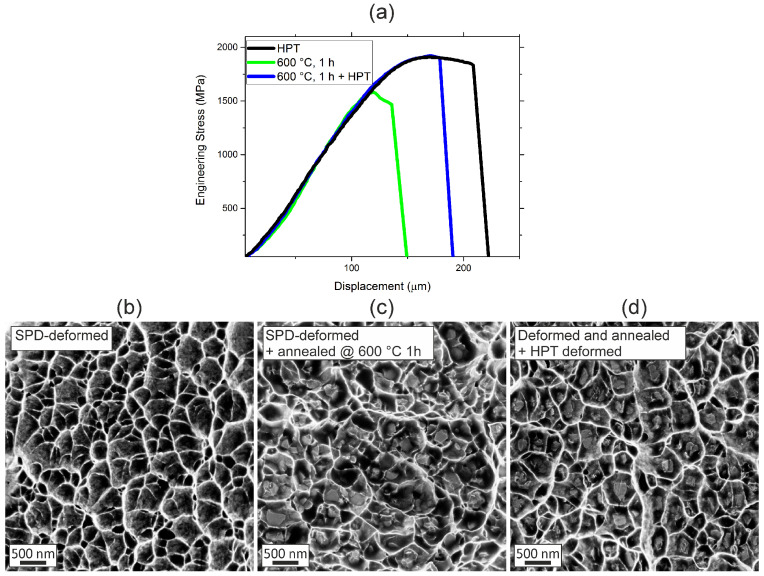
Tensile tests of the NC, the 600 °C, 1 h heat treated and the annealed and deformed sample (5 rotations) (**a**). Representative fractographs of the SPD-state (**b**), the annealed state at 600 °C for 1 h (**c**) and additionally deformed material state (**d**).

In Figure 9, the impact of the annealing treatment and the subsequent deformation for 5 rotations on the tensile properties is presented. The NC state has a relatively high yield strength compared to coarse grained material states [2], but due to the low work-hardening rate, the uniform elongation is limited (Figure 9a). A typical fractography exhibits a dimple structure with diameters much larger than the grain size (Figure 9b). As expected, the dimples do not exhibit any particles or inclusions that are typically responsible for void initiation in coarse-grained metallic materials [20]. Considering a gauge length of 2.5 mm and a displacement of the sample of ~150 µm, the uniform elongation is only around 6%. However, this is only an upper bound since, in the experiment, the displacement was not directly measured on the sample and is referred to as the displacement of the stroke. The annealing treatment at 600 °C for 1 h leads to a deterioration of the mechanical properties, in accordance with existing literature data; see, for example, [7]. The decreased yield or ultimate strength is a result of the grain growth and the ineffectiveness of the σ-phase particles as dislocation obstacles as discussed above. The decrease in ductility can be correlated with the typical fractography, Figure 9c, which exhibits a similar dimple structure to the SPD-state, but most dimples contain second phase particles. This implies that the additional σ-phase serves as the initiation site for void formation. The degradation of strength and ductility is restored by subsequent deformation. The increase in strength can be unambiguously explained by the deformation induced grain refinement (see Figure 7b). The improvement in ductility may have two reasons. On the one hand, the partial dissolution or fragmentation of the second phase may be favorable. However, as shown before, the dissolution is not complete, and the fractographs prove that the second particles are still present (Figure 9d). Thus, there is no crucial change in the basic failure mechanism between 600 °C and the further deformed state. On the other hand, the deformation results in increased dislocation density. From that, one may conclude that the additional phase has a minor impact on ductility. It is more likely that the change of the dislocation structure, the starvation after the first anneal, and the reintroduction of dislocations during further deformation play the more important role.

## 4. Discussion 

At first, it must be granted, that an investigation based on XRD-methods (600 °C, 1 h state) cannot unambiguously yield information regarding a dissolution on the atomic scale. In addition, very strong fragmentation could lead to a reduction in peak intensities. Atom probe tomography or TEM-EDS mappings, as performed for the 450 °C anneals, would have been more suitable. Nevertheless, the SEM investigations on the 600 °C and image analyses point in the direction of a reduction of the σ-phase fraction as well as also findings in the literature: 

The limited re-dissolution reported in both cases of the 450 °C, 15 h, and 600 °C, 1 h, annealing states is surprising considering previous publications on this topic. Firstly, Lee et al. [10] reported a significant re-dissolution of second-phase particles (especially the B2-FeCo-rich phase as well as the L_10_ NiMn-rich phase) during nanoindentation, where plastic strains are comparably small compared to the strains applied via HPT. Secondly, it was shown recently that a single-phase disordered fcc state can be produced by mechanically alloying an elemental powder mix of the CrMnFeCoNi alloy at low temperatures [21]. 

In principle, the deformation and intermixing mechanism in multi-phase materials during HPT processing depends strongly on multiple parameters, including the yield-strength difference of the constituent phases, their volume fraction, shape, and initial size, as well as the strain path [22]. Considering these parameters, the different results by Kilmametov et al. [21] starting from elemental powders is reasonable, since especially the yield strength difference between the constituent phases in the present study is very high, especially considering the hard intermetallic phases (L_10_-NiMn, σ). However, it also has to be noted that unlike ball milling, where alloying elements must co-deform in order to achieve intermixing, it was shown that co-deformation is not necessary to induce mechanical mixing in the HPT process [22,23,24].

Other studies of phase dissolution in systems with hard and soft constituents showed, that the mechanical mixing process at intermediate strains is dominated by a fracture and fragmentation of the hard phases and an abrasion-like process of these phase [25] However, the effectiveness of this fracture and fragmentation process decreases if the particle size decreases and if the circularity of the particles increases, which also influences the abrasion process in the hard phase [22]. The differing re-dissolution behavior of the 450 °C, 15 h, and the 600 °C, 1 h, annealing states (no significant re-dissolution vs. partial re-dissolution) can be partly explained by the fact that the initial particle size is quite different. Furthermore, it is often argued that mechanical re-dissolution is aided by an increasing interface energy between matrix and particles due to dislocation accumulation [10,24,26]. In the 600 °C, 1 h annealing state, grains are significantly larger than in the 450 °C, 15 h state, allowing for a larger dislocation pile-up and hence a higher interface energy, while in the NC microstructure seen for the 450 °C, 15 h annealing state, dislocation accumulation should be very limited to the small grain size. In consideration of the grain size of the σ-phase after deformation, in the 600 °C, 1 h annealing state, a relatively low volume fraction of the σ-phase is present in the annealed material, which leads to diminished contact between the hard particles flowing in the softer matrix. This is considered disadvantageous for the fragmentation process [25]. For this reason and the limited co-deformation, caused by the high strength of the σ-phase, the refinement of the hard phase in the 600 °C, 1 h annealing state is minimal. 

In case of the 450 °C, 15 h annealing state also the brittleness of the material has to be considered. Although the HPT process is known to be able to deform brittle materials even at room temperature, cracking occurs in these samples during unloading. On the other hand, deformation localization, which also leads to crack formation, is often detrimental to achieving homogenous intermixing. 

Regarding tensile ductility, no improvement was found with the introduction of a second phase. However, this result still does not mean that the multi-phase concept in the nanocrystalline Cantor alloy should be withdrawn. Firstly, as also shown in the present study, ductility can be improved by post-deformation (Figure 9a). Secondly, for demands such as thermal stability, wear resistance, or fatigue when short crack effects are considered, the multi-phase state may still be more favorable. 

## 5. Conclusions

In summary, the concept of microstructure engineering by post-annealing deformation in the nanocrystalline Cantor-alloy in order to achieve a mechanically more favorable microstructure state by re-dissolution and redistribution of the hard second phases is challenging. In case of the 600 °C, 1 h annealing state, re-dissolution of a significant amount of σ-phase was possible, but a full re-dissolution is likely only possible with much higher applied strains. As for the 450 °C, 15 h state, the main challenge is the high inherent brittleness of the sample and the considerable gap in yield-strength between the constituent phases, and both the formation of cracks and shear localizations can lead to poor re-dissolution processes. However, in both cases, a better re-dissolution can likely be achieved if a better co-deformation of phases is accomplished, which should be possible by employing HPT processing at elevated temperatures. 

## Figures and Tables

**Table 1:** Summarized results of the phase quantification of the 450 °C, 15 h based on STEM-EDS images. Additional results for 600 °C, 1 h sample after annealing and after 5 rotations by HPT based on point analysis of BSE-images. Numbers are given in vol%.

	450 °C, 15 h			
	B2-FeCo	A2-Cr	L_10_-NiMn	fcc-Cantor Alloy
As-annealed	17.5 ± 2	17.3 ± 2	20.2 ± 2	bal.
After 5 rotations	20.2 ± 4	19.8 ± 2	15.7 ± 4	bal.
	600 °C, 1 h			
	σ		fcc-Cantor alloy	
As-annealed	19.5 ± 1		bal.	
After 5 rotations	13.6 ± 2		bal.	

## Data Availability

The data presented in this study are available on request from the corresponding author. The data are not publicly available due to ongoing research activities.
